# Perceived maternal disapproval of friends: How mothers shape and respond to child and friend adjustment problems

**DOI:** 10.3389/fpsyg.2022.1015506

**Published:** 2022-12-16

**Authors:** Goda Kaniušonytė, Rita Žukauskienė, Aistė Bakaitytė, Brett Laursen

**Affiliations:** ^1^Department of Psychology, Mykolas Romeris University, Vilnius, Lithuania; ^2^Department of Psychology, Florida Atlantic University, Fort Lauderdale, FL, United States

**Keywords:** friendship, peer influence, emotional problems, conduct problems, maternal disapproval of friends, actor-partner interdependence model

## Abstract

The present study examines relations between adjustment problems and perceptions of maternal disapproval of friends in a sample of Lithuanian public middle-school students. The participants (ages 10 to 14) were 284 children (148 boys, 136 girls) who were involved in 142 stable friendships. Each friend described their own conduct problems, emotional problems, and perceptions of maternal disapproval of friends twice during the same academic year (*M* = 14.4 weeks apart). Dyadic analyses replicated previous findings in that one friend’s conduct and emotional problems forecast changes in the same problems in the other friend. Greater initial problems also anticipated increases in children’s’ perceptions of disapproval of friends by their own—but not their friend’s—mother, highlighting maternal efforts to manage the relations of troubled children. These efforts met with mix success. On the one hand, maternal disapproval of friends did not result in subsequent declines in adjustment problems among their own children; to the contrary, the conduct problems of boys increased with greater maternal involvement. On the other hand, adjustment problems declined among children whose friends reported higher levels of maternal disapproval, suggesting that maternal friendship management may interfere with the spread of problems between children.

## 1. Introduction

Parents worry about peer influence. And for good reason: During the pre-and early adolescent years, friends come to rival and even surpass parents in companionship, closeness, and importance (Laursen and Williams, 1997). Friend influence over problem behavior also grows during this period. Meta-analytic results revealed small but consistent influence effects, indicating that friends become more similar on a host of adjustment (i.e., conduct and emotional) problems (Giletta et al., 2021). Not surprisingly, many parents respond to these developments by attempting to direct the peer relationships of their children. In one survey, almost two-third of parents reported that they were actively involved in attempts to manage these relationships (Furstenberg et al., 1999). Although parental monitoring appears to have a salutatory effect (Lippold et al., 2014), active involvement, such as disapproval or prohibition of peer relations, can backfire, with undesirable consequences (Keijsers et al., 2012). The present study seeks to replicate and extend previous work on the topic, to explore the antecedents and consequences of maternal disapproval of friends. Specifically, we examined whether child reports of maternal disapproval of friends anticipate changes in their own emotion and behavior problems and in those of their best friend. We also examined whether mothers increased friendship management efforts in response to their own child’s behavior problems and to those of their child’s best friend. We focus on the early middle-school years, when children spend increasing amounts of time with friends, outside of direct parent supervision (Santos and Vaughn, 2018).

Parents may influence their children through direct and indirect means. We know quite a bit about indirect practices, such as parental connectedness, which has a positive effect on child functioning, through its contribution to self-esteem (Huey et al., 2020), and parent psychological control, which has a detrimental impact on peer relations through emotional dysregulation (Dickson et al., 2019). Far less is known about direct parent efforts to manage friendships. One frequently adopted strategy to manage peer relationships involves condemning, restricting, or prohibiting access to certain friends, in an attempt to limit their perceived nefarious impact (Tilton-Weaver and Galambos, 2003). Such practices yield mixed results. One study suggests that parents have some success in guiding children toward the selection of well-adjusted friends (Mounts, 2000). Another indicated that parent management of friendships was tied to increased delinquent activity (Mounts, 2001). Other studies indicate that friend disapproval and prohibition foster increases in delinquency (Tilton-Weaver and Galambos, 2003), behavior problems (Xiong et al., 2020), and defiance against parental rules (Vansteenkiste et al., 2014). Underscoring the “forbidden fruit” effect are results suggesting that parent-reported prohibition of friendships predicted heightened delinquent activity through increased contact with deviant peers (Keijsers et al., 2012).

Conclusions from prior research must be tempered by statistical concerns. Traditional parametric statistics are inappropriate for nonindependent dyadic data, because correlated partner reports violate assumptions of independence, bias error estimates, and compromise statistical tests (Kenny, 1996). The Actor-Partner Interdependence Model (APIM: Kashy and Kenny, 2000) was designed to overcome these obstacles, partitioning variance shared across partners on the same variables from variance that uniquely describes within- and between-partner associations. The present study is unique in that it utilizes a longitudinal framework to examine the bidirectional interplay of friend adjustment problems and perceptions of maternal friendship management across a 3–4 month period in a sample of early middle-school friends. Bivariate dyadic analyses will take advantage of data describing both friends and both friends’ mothers at both time points.

We expected to replicate previous findings of friend influence over conduct problems and emotional problems (Giletta et al., 2021), such that greater levels of adjustment problems on the part of one friend should lead to increases in the same on the part of the other friend. Consistent with results from previous studies, we expected that mothers would respond to adjustment problems in their own children by increasing efforts to manage friendships (Tilton-Weaver and Galambos, 2003). We suspected that mothers would express disapproval when confronted with adjustment problems among the friends of their children, but given the level of ignorance that many parents have about the affiliates and activities of their offspring (e.g., Yang et al., 2006), we were not confident that reliable associations would emerge. We did not advance specific hypotheses about the adjustment consequences of maternal disapproval of friends. Clearly, parents intend for their efforts to mitigate child problem behaviors, but some studies find that they have the opposite effect (Keijsers et al., 2012; Xiong et al., 2020). Should mothers succeed in disrupting interactions with friends, then we would expect that the friends of their children would benefit; here too, we recognized the possibility of iatrogenic effects.

Moderators were considered. Previous studies have identified sex differences in the degree to which friends influence one another (Giletta et al., 2011) and in parent efforts to interfere in peer relationships (Tilton-Weaver and Galambos, 2003), so child sex was examined as a potential moderator of patterns of association.

## 2. Materials and methods

### 2.1. Participants

Participants included 284 (148 boys, 136 girls) students attending three middle schools in a mid-sized community in Lithuania. The sample consisted of 94 5th graders (*M* = 10.87 years, SD = 0.39), 84 6th graders (*M* = 11.85 years, SD = 0.43), and 106 7th graders (*M* = 12.75 years, SD = 0.45). Of this total, 199 lived with both biological parents, 44 lived in single-parent households, 39 lived in step-parent households, and 4 had other living arrangements (e.g., grandparents). Nearly all families were ethnic Lithuanian.

Middle schools (grades 5 through 8) ranged in size from 205 to 621 (*M* = 383). Classes in these middle schools ranged in size from 14 to 31 (*M* = 26). The typical school day begins at 8 AM and ends at 3 PM. Middle-school students in Lithuania keep the same classmates during the course of a school day.

### 2.2. Procedure

Written parent consent and student assent were required for participation. Letters of invitation were sent home to parents of all students in 33 classrooms; 65.7% (*N* = 756) elected to participate. Trained research assistants administered surveys on computer tablets to students during regular school hours in the Fall and Spring of the 2021–2022 academic year (*M* = 14.4 weeks apart). The project was approved by school officials and the university ethics committee [6/-2020].

Of the 692 students who participated in both waves of data collection, 590 reported at least one reciprocated friendship at the outset, and 470 reported reciprocated friendships that were stable across both time points. Some students participated in more than one stable friendship (*M* = 1.77, SD = 1.22). In these cases, analyses were restricted to higher-ranked friends. A total of 184 participants were eliminated because the other member of the dyad was involved in a different reciprocated friendship. There were no greater-than-chance differences in any study or demographic variable between the final sample and students who were eliminated and students who only participated in one wave of data collection.

Students could skip items they did not wish to answer. For the variables included in the study, an average of 8.3% (Range = 4.2%–15.5%) of variables had missing responses in the Fall and an average of 13.5% (Range = 9.2%–21.5%) had missing responses in the Spring. Little’s MCAR test indicated that data were missing completely at random, *χ*^2^(2,479) = 2341.96, *p* = 0.976. Item-level missing data were imputed using an EM algorithm with 25 iterations. There were no wave level missing data because participation in a stable friendship was a precondition for inclusion in the study.

### 2.3. Measures

*Friendships.* Participants identified and rank-ordered up to five friends (“Who are your friends?”) from a roster that included all children in the classroom. Stable reciprocated friends were defined as dyads in which both partners nominated each other as friends at both time points. The overwhelming majority (97.2%) of stable friend dyads were the same sex. Of the 142 stable, reciprocated friendships included in the analyses, 51.1% involved top-ranked friends, 21.3% involved 2^nd^ ranked friends, and 27.6% involved lower-ranked friends.

#### 2.3.1. Adjustment problems

Participants completed an abbreviated version of the Strengths and Difficulties Questionnaire (Goodman, 1997). Items were rated on a scale ranging from 1 (*never*) to 5 (*always*). Item scores were averaged. *Emotional problems* include 6 items (*α* = 0.82–0.85) that measure anxiety and sadness (e.g., I worry a lot). *Conduct problems* include 5 items (α =0.69–0.75) that measure disruptiveness and nonconformity (e.g., I fight a lot.).

#### 2.3.2. Maternal disapproval of friends

Participants completed 5-items from the Parental Management of Peers Inventory (Mounts, 2000), which assessed perceptions of maternal disapproval of or prohibition of peer relationships (e.g., My mother tells me if she does not want me to hang around with certain kids.). Items were rated on a scale ranging from 1 (*never*) to 5 (*always*). Item scores were averaged. Internal reliability was acceptable (*α* = 0.85–0.87).

#### 2.3.3. Potential confounding variables

To isolate effects to our target constructs, we conducted supplemental analyses that included, as Fall covariates, variables known to correlate with maternal friendship management and child adjustment difficulties. Participants completed two measures of parenting practices, including 3 items (Kerr et al., 2010) that assessed *Maternal Behavioral Control* (e.g., Do you need your mother’s permission to stay out late on a weekday evening?) and 5 items (Barber et al., 2005) that assessed *Maternal Psychological Control* (e.g., *My mother is less friendly with me if I do not see things her way.).* Participants also completed a short version of Network of Relationships Inventory (Furman and Buhrmester, 1985), which included an 8-item measure of *Maternal Support* (e.g., My mother likes or approve of the things I do.) and a 4-item measure of. *Maternal Negativity* (My mother and I get mad or upset with each other.). Internal reliabilities were acceptable (*α* = 0.82–0.94).

All items for all variables are listed in Supplementary Table S1.

### 2.4. Plan of analysis

**Figure 1:**
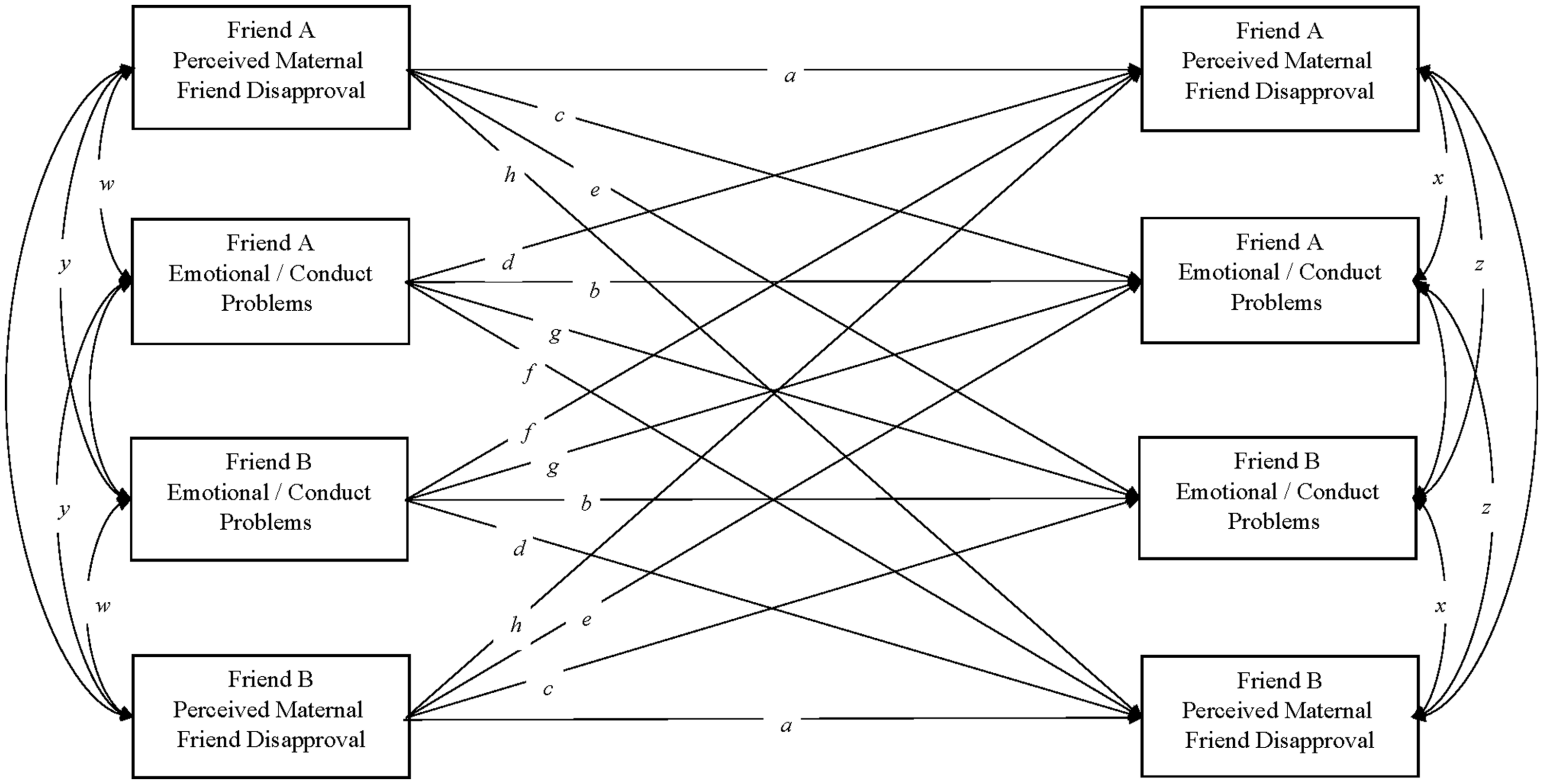
Measurement model of bivariate longitudinal indistinguishable dyad APIM. The model includes stability paths (*a* and *b*), within-person influence paths (c and d), between-person influence paths (*e*, *f*, *g*, and *h*), within-person correlations (*w* and *x*) between-person correlations (*y* and *z*). Identical label reflects equality constraints.

Indistinguishable Actor-Partner Interdependence Model analyses modified for longitudinal data (Laursen et al., 2011) were conducted using Mplus 8.4 (Muthén and Muthén, 1998–2018) with Robust Maximum Likelihood (MLR) estimation. Figure 1 depicts the fully saturated longitudinal APIM. Equality constraints reflect interchangeable partner positions (Olsen and Kenny, 2006). Within-dyad constraints were imposed on within-individual stability paths (actor paths *a* and *b*), within-individual influence paths (actor paths *c* and *d*), between-individual influence paths (actor paths *e*, *f*, *g,* and *h*), within-individual correlations (*w* and *x*), and between-individual correlations (*y* and *z*), as well as on the means (*m* and *n*) and variances (*v* and *u*) of Time 1 scores, and the intercepts and residuals of Time 2 scores.

To obtain fit indexes with an otherwise fully saturated model, we included Fall emotional problems as a Time 1 covariate in the conduct problems model and Fall conduct problems as a Time 1 covariate in the emotional problem model. The same pattern of results emerged when covariates were omitted. Standard model fit indices were applied. Acceptable model fit requires a nonsignificant (*p* > 0.05) Chi-square test; the root-mean-square error of approximation (RMSEA) and standardized root-mean-squared residual (SRMR) should be 0.06 or lower; the Tucker–Lewis index (TLI) should exceed 0.95 (Kline, 2016).

Multiple group analyses were performed to examine sex (boys vs. girls) and household structure (two biological parents vs. all other households) differences in cross-lagged paths. No differences for household structure emerged. Statistically significant (*p* < 0.05) sex differences are reported in the final model. Demographic variables (age, grade, and friendship ranking) and potential confounding variables (maternal behavioral control, maternal psychological control, maternal support, and maternal negativity) were separately entered into the model as Time 1 covariates. The same pattern of statistically significant results emerged. The results presented included the 4 other-sex friend dyads; the same pattern of statistically significant results emerged when they were omitted.

## 3. Results

### 3.1. Preliminary analysis

Intraclass correlations (interpreted as *r*^2^) revealed borderline statistically significant (*p* < 0.10) associations between partner reports on conduct problems (intraclass *r* = 0.18–0.23) and statistically significant (*p* < 0.05) associations between partner reports on emotional problems (intraclass *r* = 0.28–0.33).

Given that friend reports were not independent, bivariate correlations and ANOVAs were conducted with one randomly selected member of each dyad. The same pattern of results emerged in analyses with the other member of the dyad.

Table 1 presents intercorrelations. Conduct problems were correlated with emotional problems and perceived disapproval of friends at both time points (*p* < 0.05). All autocorrelations were statistically significant.

**Table 1:** Intraindividual interclass correlations, means and standard deviations.

Variable	1	2	3	*M*	(SD)
1. Emotional problems	0.70** [0.58, 0.79]	0.38** [0.21, 0.54]	0.16 [−0.04, 0.35]	2.36	(0.69)
2. Conduct problems	0.52** [0.39, 0.64]	0.58** [0.41, 0.73]	0.18* [0.03, 0.32]	1.80	(0.59)
3. Perceived maternal friend disapproval	0.14 [−0.03, 0.31]	0.24** [0.06, 0.43]	0.53** [0.38, 0.67]	2.28	(1.03)
*M* (SD)	2.43 (0.85)	1.81 (0.63)	2.23 (1.07)		

*N* = 142 randomly selected individuals from each friend dyad. 95% Confidence intervals are presented in brackets. Autocorrelations are presented on the diagonal. Intercorrelations at Fall are above the diagonal. Intercorrelations at Spring are below the diagonal. Variables were rated on a scale from 1 (never) to 5 (always).

*p < 0.05, **p < 0.01, two-tailed.

Separate 3 (grade) × 2 (sex) × 2 (time) repeated-measures ANOVAs were conducted with emotional problems, conduct problems, and perceived disapproval of friends as dependent variables. There were no statistically significant main effects. There was a two-way grade x time interaction on perceived disapproval of friends, *F*(1, 132) = 4.37, *p* = 0.014). Follow-up *t*-tests revealed that maternal disapproval of friends significantly decreased over the course of the school year among 7^th^ graders (*d* = 0.37), but not among 5^th^ (*d* = 0.26) or 6^th^ graders (*d* = 0.17).

### 3.2. Longitudinal associations between emotional problems and perceived maternal disapproval of friends

**Figure 2:**
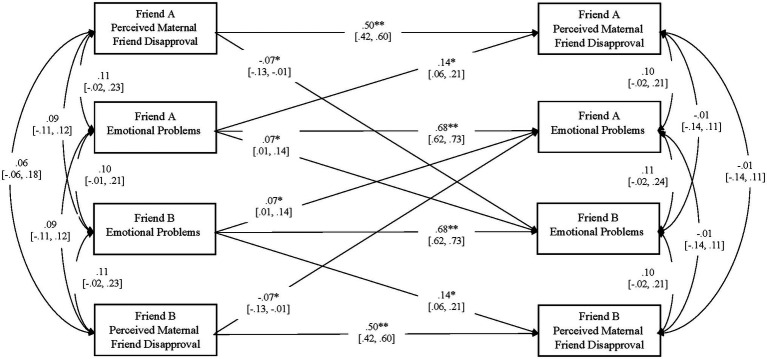
Longitudinal associations between friend internalizing symptoms and perceptions of material disapproval of friends. *N* = 142 dyads. For each path, 95% confidence intervals are presented in brackets. **p < 0.01,*p < 0.05, two-tailed.

Figure 2 presents results for emotional problems. The model fit the data, *χ*^2^(30) = 25.51, *p* = 0.70, TLI = 1.000, RMSEA = 0.000 [0.000, 0.035], SRMR = 0.051.

Fall emotional problems were positively associated with changes in partner emotional problems from Fall to Spring. Higher levels of one friend’s emotional problems predicted increases in the other friend’s emotional problems. Fall emotional problems were also positively associated with changes in self-perceptions of maternal disapproval of friends from Fall to Spring. Higher initial levels of one friend’s emotional problems predicted increases in the same friend’s reports of maternal disapproval of friends. Finally, Fall maternal disapproval of friends was negatively associated with changes in partner emotional problems from Fall to Spring. Higher initial levels of one friend’s maternal disapproval of friends predicted decreases in the other friend’s emotional problems.

### 3.3. Longitudinal associations between conduct problems and perceived maternal disapproval of friends

**Figure 3:**
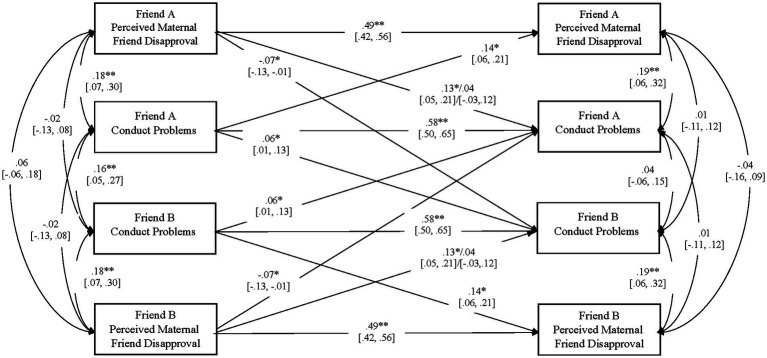
Longitudinal associations between friend externalizing symptoms and perceptions of material disapproval of friends. *N =* 142 dyads. For each paths, 95% confidence intervals are presented in brackets. Values to the left of the slash represent paths for boys and values to the slash represent paths for girls. **p < 0.01, *p < 0.05, two-tailed.

Figure 3 presents results for externalizing symptoms. The model fit the data, *χ*^2^(30) = 20.21, *p* = 0.91, TLI = 1.000, RMSEA = 0.000 [0.000, 0.018], SRMR = 0.047.

Fall conduct problems were positively associated with changes in partner conduct problems from Fall to Spring. Higher levels of one friend’s conduct problems predicted increases in the other friend’s conduct problems. Fall conduct problems were also positively associated with changes in self-perceptions of maternal disapproval of friends from Fall to Spring. Higher initial levels of one friend’s conduct problems predicted increases in the same friend’s reports of maternal disapproval of friends. Fall maternal disapproval of friends was negatively associated with changes in partner conduct problems from Fall to Spring. Higher initial levels of one friend’s maternal disapproval of friends predicted decreases in the other friend’s conduct problems. Finally (for boys only), Fall perceptions of maternal disapproval of friends were positively associated with changes in self-reports of conduct problems from Fall to Spring. Higher initial levels of one friend’s perceived maternal disapproval of friends predicted increases in the same friend’s conduct problems.

## 4. Discussion

The present study examined (a) whether friends influence one another’s behavior problems and (b) the degree to which behavior problems affect and are affected by maternal disapproval of friends. Three major findings emerged. First, replicating previous findings (e.g., Havewala et al., 2021), we found that emotional problems and conduct problems spread between friends. Second, emotional problems and conduct problems elicited increases in child but not friend reports of maternal disapproval of friends. Third, perceived maternal disapproval of friends had differing effects on child adjustment. Child reports of friendship management predicted increases in their own conduct problems (for boys only) but decreases in their friend’s emotional problems and conduct problems.

As expected, adjustment problems spread between friends over the course of the school year. The findings are consistent with well-documented claims about pervasive friend influence during late childhood and early adolescence (Giletta et al., 2021). Friend influence manifests itself through a variety of mechanisms, including imitation, cooperation, and identity signaling (Laursen and Veenstra, 2021). Friends model and express behavioral preferences, reward conformity, and punish nonconformity. Influence serves an important role in friendships, increasing similarity between partners, which serves to promote the stability of the relationship by increasing shared rewards and reducing conflict (Laursen, 2017).

We know surprisingly little about maternal efforts to manage the friendships of children. The present study is a first step toward understanding how these efforts shape and are shaped by the behaviors of children. As expected, mothers responded to their own child’s emotional and conduct problems with increased efforts to manage friendships, presumably because mothers ascribe these problems to affiliation with untoward peers. Parents adjust practices according to perceptions of child characteristics (Kerr and Stattin, 2003). Findings from a prior concurrent correlational study indicate that parents of 6^th^ and 9^th^ grade adolescents both seek information about friends and convey disapproval of them when children report (a) high levels of their own behavior problems and (b) high levels of their friends’ behavior problems (Tilton-Weaver and Galambos, 2003). Our longitudinal findings confirmed the former but not the latter. We can only speculate as to why we did not find both. Of course, the most parsimonious explanation is that the prediction of change (the definition of influence) is a much higher bar that identifying concurrent correlations. It may simply be that friend problems are not strong indicators of parent behavior, particularly since parent knowledge of adolescent friends is far from perfect (Stattin and Kerr, 2000) and adolescent reports of friend delinquent activities are far from accurate (Belendiuk et al., 2010). Note also that our sample described youth during the transition into adolescence. It may be that parents are more active in their management attempts as adolescents get older and their behavior problems become more apparent. The most prudent conclusion, absent further research, is that parental disapproval of friendship is more apt to arise in response to child problems than friend problems.

How effective are maternal efforts to manage the friendships of their children? Seemingly paradoxical findings emerged. Disapproval of friends had no discernable impact on child emotional problems or on the conduct problems of daughters. Among sons, however, conduct problems increased as a result of maternal disapproval of friends. Others have reported similar findings, with parental friendship prohibition at the beginning of 9^th^ grade predicting increases in delinquent behavior by the end of the school year (Mounts, 2001). The latter findings were interpreted as rebellion to perceived parent meddling, which has the knock-on effect of promoting deviant behavior (Keijsers et al., 2012). Findings from friends offer some (tentative) insight. Adolescent reports of perceived maternal disapproval of friends predicted decreases in best friend emotional and conduct problems, suggesting that mothers may be disrupting the spread of problem behaviors by interfering with friend contact. Adolescents may be spending less time with or engage in less problematic behavior with those friends, which helps the friend but has an iatrogenic effect on sons. We await new research that sheds light on this dynamic.

Our study is not without limitations. First, participants could only nominate classmates as friends. Out-of-class friends were not included, which may have diminished the magnitude of the effects given that delinquent adolescent tend to find delinquent mates in nonclass settings (Persson et al., 2007). High-quality friendships tend to be more influential than low-quality friendships and more satisfied partners tend to be more susceptible to influence than less satisfied partners (Hiatt et al., 2017), in keeping with suggestions that friend influence effects are not distributed equally between or within dyads (Laursen and Faur, 2022). It is not clear whether or how the quality of a friendship might alter parent efforts to manage the affiliation. Second, we focused on adolescent reports of maternal friend disapproval; the absence of mother reports raises the prospect of shared reporter variance bias in actor effects (only). Mothers and adolescents differ in their perceptions of maternal friendship management (Mounts, 2007), although a strong case can be made that the impact of parenting depends more on how the adolescent perceives and interprets parent actions than on how parents report them (Stattin and Kerr, 2000). Parent management of friendship may also depend on parent perceptions of child behavior problems. Visible behaviors, like conduct problems, contain more overlap between parent and child reporters and have stronger associations with parenting behaviors, than less visible behaviors like emotional problems (Valdes et al., 2016). We employed indistinguishable dyad analyses, which fix effects to be equal across partners. This is a blunt instrument. Distinguishable dyad analyses may well reveal individual differences in patterns of association, shedding light on who influences whom. Finally, it is worth noting that the participants attended school in a small, homogeneous Northern European community. Child development in Lithuania resembles that in other homogeneous European communities (Kaniušonytė and Žukauskienė, 2018), although some of the mothers in the current study were raised when the country was part of the Soviet Union, during a time when obedience was a priority (Gorlizki and Khlevniuk 2020). Most Eastern European countries still report higher levels of “traditional” parenting, compared to their Western European counterparts (Maslauskaitė and Steinbach, 2020). The extent to which traditional parenting encompasses friendship disapproval is not clear. It remains to be seen whether the findings generalize to youth living in heterogeneous, urban contexts, particularly those in non-Western settings.

The findings confirm that friends influence one another’s problem behaviors. Mothers respond to these problems with attempts to shape peer relationships. These efforts meet with mixed success, having little or no impact on the behavior of their own children (except when they make things worse), but sparing the friends of their children from the spread of problems. To be sure, this is not what parents have in mind when they attempt to manage the friendships of their children. Intervention efforts should focus on constructive, effective strategies at reducing behavior problems, given that blaming problems on peers is benign at best and counterproductive at worst.

## Data availability statement

The raw data supporting the conclusions of this article will be made available by the authors, without undue reservation.

## Ethics statement

The studies involving human participants were reviewed and approved by Committee of Psychological Research Ethics, Institute of Psychology, Mykolas Romeris University. Written informed consent to participate in this study was provided by the participants’ legal guardian/next of kin.

## Author contributions

GK: conceptualization, methodology, formal analysis, investigation, and writing—original draft preparation. RZ: investigation, writing—review and editing, project administration, and funding acquisition. AB: software, investigation, data curation, and writing—review and editing. BL: conceptualization, methodology, supervision, and writing—review and editing. All authors contributed to the article and approved the submitted version.

## Funding

This project has received funding from European Regional Development Fund (project No 09.3.3-LMT-K-712-71-0009) under grant agreement with the Research Council of Lithuania (LMTLT).

## Conflict of interest

The authors declare that the research was conducted in the absence of any commercial or financial relationships that could be construed as a potential conflict of interest.

## Publisher’s note

All claims expressed in this article are solely those of the authors and do not necessarily represent those of their affiliated organizations, or those of the publisher, the editors and the reviewers. Any product that may be evaluated in this article, or claim that may be made by its manufacturer, is not guaranteed or endorsed by the publisher.

## Supplementary material

The Supplementary material for this article can be found online at: https://www.frontiersin.org/articles/10.3389/fpsyg.2022.1015506/full#supplementary-material
