# Supplementary material for: Perceived maternal disapproval of friends: How mothers shape and respond to child and friend adjustment problems
Source: Front Psychol. 2022 Dec 16;13:1015506. doi: 10.3389/fpsyg.2022.1015506 (PMC9800894; doi:10.3389/fpsyg.2022.1015506)
Supplement: Supplementary file 1 [file Table_1.DOCX]

Supplementary Material

# Supplementary Tables

**Table S1**. Questionnaire items

|  | **Adjustment problems** |
| --- | --- |
|  | ***Emotional problems*** |
| **Q1** | I get a lot of headaches or stomachaches |
| **Q2** | I worry a lot. |
| **Q3** | I am often unhappy or sad or empty |
| **Q4** | I have many fears, I am easily scared |
| **Q5** | I am nervous in new situations. I easily lose confidence (wording in Lithuanian: New environment frightens me) |
|  | ***Conduct problems*** |
| **Q1** | I break rules at home, school, or elsewhere. |
| **Q2** | I fight a lot. |
| **Q3** | Others accuse me of lying or cheating. |
| **Q4** | I take things that are not mine (from home, school or elsewhere). |
| **Q5** | I get very angry, and often lose my temper |
|  | **Maternal disapproval of friends** |
| **Q1** | My mother tell me if she don’t want me to hang around with certain kids. |
| **Q2** | My mother tell me that she don’t approve of the things my friends do. |
| **Q3** | My mother let me know who she wants to be my friends. |
| **Q4** | My mother want me to be friends with kids who are good students. |
| **Q5** | My mother tell me that she don’t like my friends. |
|  | **Potential Confounds** |
|  | ***Psychological control*** |
| **Q1** | If I have hurt my mothers feelings, she stops talking to me until I please her |
| **Q2** | My mother brings up my past mistakes when she criticizes me |
| **Q3** | My mother changes the subject whenever I have something to say |
| **Q4** | My mother Often interrupts me. |
| **Q5** | My mother is less friendly with me if I do not see things her way. |
|  | ***Behavioral control*** |
| **Q1** | I need to have my mothers permission to stay out late on a weekday evening. |
| **Q2** | I have to ask my mother before I can make plans to do something on a Saturday night. |
| **Q3** | My mother always make me tell her where I am at night, who I am with, and what we are doing together. |
|  | ***Maternal social support*** |
| **Q1** | My mother and I hang around and have fun together. |
| **Q2** | My mother and I help each other out. |
| **Q3** | My mother and I really care about each other. |
| **Q4** | My mother and I go places and do enjoyable things together. |
| **Q5** | My mother and I like or love each other. |
| **Q6** | My mother treat me like I am admired and respected. |
| **Q7** | My mother like or approve of the things I do. |
| **Q8** | My mother treat me like I'm good at many things. |
|  | ***Maternal negativity*** |
| **Q1** | My mother and I disagree about many things. |
| **Q2** | My mother and I argue with each other. |
| **Q3** | My mother and I get annoyed with each other. |
| **Q4** | My mother and I get mad or upset with each other. |
